# Early results of aortic root reoperation

**DOI:** 10.1186/1749-8090-8-S1-P4

**Published:** 2013-09-11

**Authors:** T Gomibuchi, T Takano, K Komatsu, Y Ohtsu, T Terasaki, Y Wada, T Seto, D Fukui, J Amano

**Affiliations:** 1 Cardiovascular Surgery, Shinshu University, Matsumoto, Japan

## Background

Reoperation of aortic root is considered challenging because of technical difficulty and relatively low incidence of surgical indications. It is still not clear whether the redo root surgery could be performed safely although the primary Bentall procedure is a safe and established operation for aortic root surgery. We evaluated early outcomes of re- aortic root replacement comparing to primary Bentall operation.

## Methods

From 1996 to 2012, we operated 43 patients for Aortic root replacement. Among these 42 patients, 11 patients underwent re-aortic root replacement (group R), whereas 31 patients underwent primary Bentall procedure (group B). We retrospectively investigated in-hospital mortality and operative results comparing in two groups.

## Results

The initial procedures in group R were 5 modified Bentall, 5 AVR and 1 aortic valve repair. Modified Bentall procedure was performed in 5 patients of group R, and prosthetic valve sparring root replacement (PSR) was done in 6 patients of group R. In group B we performed modified Bentall procedure in all the cases. Concomitant procedures were total arch replacement in 3 of group R and 7 in group B (p=1.0), CABG only in 4 in group B (p=0.56), and mitral valve surgery in 1 of both groups (p=0.46). One patient died in each group, therefore, the in-hospital mortality was 9.1 % and 3.2 % in group R and B, respectively (p= 0.46). Piehler’s modification was used significantly frequent in group R for coronary artery reconstruction (R; 36.4%, B; 0%, p=0.003). Operation time was relatively longer in group R (R; 690±326min, B; 520±162min, p=0.065) although CPB time (R; 337±225min, B; 272±90min, p=0.26) and aortic closs-clamp time (R; 214±89min, B; 191±48min, p=0.35) had no difference in the two groups.

## Conclusion

Re-aortic root replacement could be performed safety although alternative technic such as PSR and Piehler’s procedure were required, which led to relatively longer operation time.

